# Dr. Harvey Parkhurst

**Published:** 1902-03

**Authors:** 


Dr. Harvey Parkhurst, U. of B. '51, of Danvers, Ill., died at his home January 16, 1902, after a very short illness, aged 79 years. He was a prominent and respected member of the medical profession.

